# Extent of differential allelic expression of candidate breast cancer genes is similar in blood and breast

**DOI:** 10.1186/bcr2458

**Published:** 2009-12-10

**Authors:** Ana-Teresa Maia, Inmaculada Spiteri, Alvin JX Lee, Martin O'Reilly, Linda Jones, Carlos Caldas, Bruce AJ Ponder

**Affiliations:** 1 Cancer Research UK Cambridge Research Institute, Li Ka Shing Centre and Department of Oncology, University of Cambridge, Robinson Way, Cambridge CB2 0RE, UK; 2 Cambridge Experimental Cancer Medicine Centre, Li Ka Shing Centre, Robinson Way, Cambridge CB2 0RE, UK; 3 Strangeways Research Laboratory, Department of Oncology, University of Cambridge, Worts Causeway, Cambridge, CB1 8RN, UK; 4 Translational Cancer Therapeutics Lab, Cancer Research UK London Research Institute, 44 Lincolns Inn Fields, London WC2A 3PX, UK

## Abstract

**Introduction:**

Normal gene expression variation is thought to play a central role in inter-individual variation and susceptibility to disease. Regulatory polymorphisms in cis-acting elements result in the unequal expression of alleles. Differential allelic expression (DAE) in heterozygote individuals could be used to develop a new approach to discover regulatory breast cancer susceptibility loci. As access to large numbers of fresh breast tissue to perform such studies is difficult, a suitable surrogate test tissue must be identified for future studies.

**Methods:**

We measured differential allelic expression of 12 candidate genes possibly related to breast cancer susceptibility (*BRCA1*, *BRCA2*, *C1qA*, *CCND3*, *EMSY*, *GPX1*, *GPX4*, *MLH3*, *MTHFR*, *NBS1*, *TP53 *and *TRXR2*) in breast tissue (n = 40) and fresh blood (n = 170) of healthy individuals and EBV-transformed lymphoblastoid cells (n = 19). Differential allelic expression ratios were determined by Taqman assay. Ratio distributions were compared using t-test and Wilcoxon rank sum test, for mean ratios and variances respectively.

**Results:**

We show that differential allelic expression is common among these 12 candidate genes and is comparable between breast and blood (fresh and transformed lymphoblasts) in a significant proportion of them. We found that eight out of nine genes with DAE in breast and fresh blood were comparable, as were 10 out of 11 genes between breast and transformed lymphoblasts.

**Conclusions:**

Our findings support the use of differential allelic expression in blood as a surrogate for breast tissue in future studies on predisposition to breast cancer.

## Introduction

Approximately 70% of the genetic risk associated with breast cancer is still unaccounted for and it is predicted that the remainder of susceptibility loci will include common, low-effect variants that most likely have regulatory effects. Recent genome-wide association studies (GWAS) have identified variants that account for an additional 5.9% of the genetic risk [1-5]. These variants are mostly associated with intronic and intergenic regions, with the most significant variant regulating the level of gene expression of *FGFR2 *[6]. However, as most of the identified risk loci have small effects, very large numbers of patients will have to be examined to identify further risk variants. An alternative approach for the identification of regulatory risk variants could be to use differences in allelic gene expression in heterozygotes as a quantitative phenotype [7-9].

Preferential expression from one allele is a common feature of the human genome (up to 60% of genes) and has a genetic basis [6,10-24]. Polymorphic variants at regulatory elements can cause differential allelic expression (DAE), thus using DAE as a quantitative trait could help identify such variation. The samples of choice for association studies are usually blood and saliva, however, relatively little is known about how DAE compares in multiple human tissues and it is questionable whether studying DAE in blood would be a proper surrogate for what happens in the disease target tissue. To date most DAE studies have been performed on EBV transformed lymphoblastoid cell lines (LCLs). Studies in fresh blood, liver and kidney have been reported in a small set of individuals [14,16], and one recent study looking at the expression of one gene reported that there were large tissue differences in allelic expression ratios within the same individual [25]. An analogous study has been reported in mice [26].

We aimed to perform a more extensive evaluation of differential allelic expression between blood and breast in order to assess the potential usefulness of LCL and fresh blood in association studies, to identify regulatory polymorphisms related to susceptibility to breast cancer. Here we present an analysis of DAE in 12 candidate genes (*BRCA1*, *BRCA2*, *C1qA*, *CCND3*, *EMSY*, *GPX1*, *GPX4*, *MLH3*, *MTHFR*, *NBS1*, *TP53 *and *TRXR2*) likely to be involved in breast cancer, in a large set of individuals. We compared the distribution of allelic ratios of gene expression in fresh blood (B cells and total mononuclear cells), transformed lymphoblasts, and breast tissue from unmatched healthy individuals.

## Materials and methods

### Samples

A total of 170 white cell-reduction filters from anonymous blood donors were collected from the Blood Centre at Addenbrooke's Hospital. Mononuclear cells were separated by density gradient centrifugation using Lymphoprep (Sigma, St. Louis, MO, USA), according to the manufacturer's instructions. B cells were further isolated from these samples by magnetic sorting using CD19 labelled magnetic check beads (Milteny Biotech, Bergisch Gladbach, Germany).

Normal breast tissue was collected at Addenbroke's Hospital, from 40 women undergoing aesthetic surgery, for reasons not related to cancer. All samples were analysed by a histopathologist to ensure that they were free of dysplasia. Ethical approval was obtained for the collection and research use of all blood and breast samples used in this study.

Nineteen lymphoblastoid cell lines derived from unrelated CEPH individuals were obtained from the Coriell Cell Repository. Cell lines were grown in RPMI 1640 with 10% FCS, supplemented with penicillin, streptomycin and L-glutamine, at 37°C and 5% CO_2 _(Invitrogen, Carlsbad, CA, USA).

All research was carried out in compliance with ethics guidelines and regulations. Human B cells (purified from waste products of blood donations) and normal breast samples were collected with approval from the Addenbrooke's Hospital Local Research Ethics Committee (REC reference 04/Q0108/21 and 06/Q0108/221, respectively).

### RNA, DNA and cDNA preparation

DNA was extracted from total mononuclear cells, B-lymphocytes, normal breast and lymphoblastoid cell lines by a conventional SDS/proteinase K/phenol method. Total RNA was extracted from all samples using Qiazol (Invitrogen, Carlsbad, CA, USA) following manufacturer's instructions. The RNA was subsequently treated with DNaseI and repurified using acidic phenol-chlorophorm, and ethanol precipitation.

For normal breast tissue RNA extraction, samples were soaked overnight in RNA*later*-Ice^® ^(Ambion, Austin, TX, USA), homogenised in Qiazol using the Precellys^®^24 bead mechanism (Bertin Technologies, Montigny-le-Bretonneux, France), followed by an additional centrifugation step prior to addition of chlorophorm to the lysate, to eliminate excessive fat.

cDNA was prepared from 1 μg of total RNA per 20 μl reaction using random hexamers and the Reverse Transcription kit (Applied Biosystems, Foster City, CA, USA), according to the manufacturer's instructions, and was diluted in a final volume of 100 μl.

### Genotyping

All samples were genotyped using 5' exonuclease Taqman^® ^technology (Applied Biosystems, Foster City, CA, USA). Approximately 20 ng of genomic DNA was used in a 5 μl PCR reaction constituted by Taqman^® ^master mix (Applied Biosystems, Foster City, CA, USA), the two primers, and FAM- and VIC- labelled probes, each designed to anneal specifically to either of the alleles of each single nucleotide polymorphism (SNP). After completion of the PCR, plates were analysed using the Allelic Discrimination analysis method in an ABI PRISM 7900 Sequence Detector (Applied Biosystems, Foster City, CA, USA). Genotyping was carried out in 384-well plates, with random replicates included, as well as no template controls (NTC), to ensure good quality of genotyping.

### Quantification of differential allelic gene expression

Allele specific levels of gene expression were determined in heterozygous samples using Taqman^® ^technology (Applied Biosystems, Foster City, CA, USA). Each PCR reaction contains a primer pair targeting the region surrounding the transcribed SNP (tSNP), and two probes that differ by a single nucleotide and are complementary to each of the SNP alleles. The probes are labelled with different fluorochromes (VIC and FAM), generating two signals for each sample during the real-time PCR. A standard curve was generated using a dilution series of heterozygote blood DNA, serving as a reference for the 50:50 allelic ratio. In this way, once the quantity of each allele in the different samples is extrapolated from the linear regression equation, a correction for the different background fluorescence and annealing characteristics of each probe is made automatically. We avoided using cDNA as control as we would be biasing our results towards the DAE ratio of the reference sample. In contrast, there is a perfect 50:50 ratio of the two alleles in a reference DNA sample from a heterozygote with normal chromosomal copy number.

All experiments contained replicates for each sample, and were repeated at least twice. Reactions were prepared as described for genotyping and run on an ABI PRISM 7900 Sequence Detector using the Absolute Quantification method. Ct values were obtained from ABI SDS 2.3 software (Applied Biosystems, Foster City, CA, USA) and quantities of allelic expression were extrapolated from the appropriate linear regression. We defined differential allelic expression as the log_2 _of the allelic-expression ratio calculated as log_2 _[(VIC- allele)/(FAM - allele)]. A gene was considered expressed if the PCR yielded Ct values lower than 35 cycles.

### Quantification of total gene expression

Total gene expression levels were determined in B cell cDNA samples using Taqman^® ^Gene Expression Assay pre-designed by Applied Biosystems. Results were normalized with the total levels of expression of *Actin-β*, *GAPDH*, *18S *and *β2M*.

### Statistical Analysis

Real-time PCR quantification statistics were carried out on Microsoft^® ^Excel^® ^2004 software. Percentage of variation between replicates was calculated as % var = (SD/Mean). Linear regression for Taqman^® ^standard curves was performed using the function *linest*.

All other statistical analysis was performed using the R statistical programming language [27]. For analysis of DAE in B cells a One Sample t-Test was performed to test for deviations from null hypothesis that the mean is smaller than log_2_(1.20). However, genes that presented trans-effects were analysed in absolute values with the highest expressing allele divided by the lowest one. For these genes the mean could not be used as the two sides of the distribution would cancel each other out, as explained in Results and Discussion. Furthermore, we performed variance analysis for these genes using F tests for variance. Using *MLH3 *as a reference gene with a distribution identical to DNA (no DAE), we compared all genes with trans-effect DAE.

To compare DAE across the three different tissue types, both two-sample t-test and Wilcoxon rank sum test with continuity correction were carried out, for comparing mean ratios and variances respectively.

Correlation analysis of the total level of expression vs genotype at the tSNP for these genes was performed using the Jonckheere-Terpstra test, a non-parametric test for trend among classes.

## Results

### Analysis of differential allelic expression of candidate breast cancer genes in blood cells

**Figure 1:**
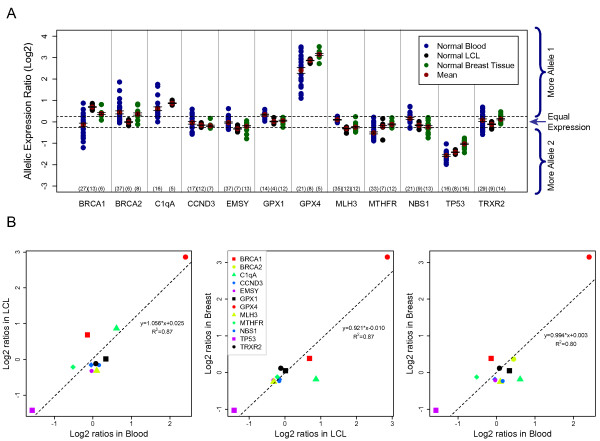
Comparison of allelic expression in blood *vs *LCL *vs *breast tissue. **(a)** Heterozygote individuals are represented as dots and are coloured blue for blood, black for LCL and green for breast tissue. The mean value for each distribution is shown as a red dot, and whiskers represent the 95% confidence interval of the mean. Dotted lines delimit the cut-off of 1.2 preferential allelic expression ratio [log_2_(1.2) = 0.263]. **(b)** Pairwise correlation analysis of the mean log_2 _allelic expression ratios for the three sample sets. Genes are identically colour coded in all three graphs. Dotted lines represent the linear regression applied to each tissue pair, and the respective equations and R^2 ^values are indicated on each graph.

We studied 12 candidate genes that are implicated in the aetiology of breast cancer (Table 1): *BRCA1*, *BRCA2*, *C1qA*, *CCND3*, *EMSY*, *GPX1*, *GPX4*, *MLH3*, *MTHFR*, *NBS1*, *TP53 *and *TRXR2 *[28-31]. Functionally, these genes are in different pathways including: DNA-damage repair, complement and coagulation cascades, cell cycle and apoptosis. For each gene, we selected single nucleotide polymorphisms (SNPs) markers in both the coding and untranslated regions (transcribed SNP or tSNP), with high heterozygosity frequency. This increased the number of informative individuals in our sample sets. To ascertain differential allelic expression we measured allele-specific transcript levels using real-time PCR Taqman^® ^technology in heterozygote samples for the selected tSNPs, and calculated the ratio of one allele versus the other (plotted as Log_2 _ratios in Figure 1a).

**Table 1:** List of coding polymorphisms investigated for differential allelic expression, with the respective possible functional effects

Gene	SNP	Aminoacid/Position	Alleles	Pathway	Functional Effect
**BRCA1**	rs799917	P871L	C/T	DSB repair	Unknown
**BRCA2**	rs144848	N372H	A/C	DSB repair	Unknown
**C1qA**	rs172378	G92G	A/G	Complement Cascade	Reduced Activity
**CCND3**	rs1051130	A259S	G/T	Cell Cycle	Unknown
**EMSY**	rs2282611	5'UTR	A/C	DNA repair	Unknown
**GPX1**	rs1050450	L200P	T/C	Antioxidant Defense	Unknown
**GPX4**	rs713041	3'UTR	C/T	Antioxidant Defense	Reduced Activity
**MLH3**	rs175080	P844L	C/T	Mismatch Repair	Unknown
**MTHFR**	rs1801133	A222V	C/T	Folate metabolism	Reduced Activity
**NBS1**	rs709816	D399D	C/T	Homologous Recombination	None
**TP53**	rs1042522	R72P	G/C	Apoptosis	Differential apoptotic potential
**TRXR2**	rs1139793	T370I	C/T	Antioxidant Defense	Unknown

DSB = double strand break.

Initial experiments on technical and biological replicates (different cDNA preparations) revealed a very good correlation between replicates and low noise/variation intrinsic to the technique ([mean allelic ratio: standard deviation] <20% and 5% for biological and technical replicates, respectively). Based on this we defined the cut-off allelic expression ratio of 1.2 for DAE presence in a heterozygote sample (indicated on Figure 1a by the dotted lines).

We started by analysing allelic expression in primary B-lymphocytes (magnetically sorted CD19^+ ^cells) from 170 unrelated healthy individuals. The aim was to first identify the genes that displayed preferential allelic expression in a homogeneous population of cells, without the possible interference of multiple cell types. We found that heterozygotes in 11 out of 12 genes (92%) showed allelic imbalances in gene expression (Table 2 and blue data points in Figure 1a). As in previous reports, we identified two patterns of differential expression. In *BRCA2, C1qA, GPX1, GPX4, MTHFR *and *TP53*, the same allele was consistently expressed at a higher level in all heterozygotes with allelic imbalance, indicating that for each of these genes the regulatory variant is in strong linkage disequilibrium (LD) with the assayed tSNP. On the other hand, in *BRCA1, EMSY, CCND3, NBS1 *and *TRXR2 *different heterozygotes preferentially expressed different alleles. In this case, expression is likely to be controlled by cis-acting elements that are not in strong linkage disequilibrium with the tSNP.

**Table 2:** Differential allelic expression ratios in fresh B cells

Gene	SNP	Heterozygotes with DAE		Mean DAE	SD	Min-Max
**BRCA1**	rs799917	T>C	44% (12/27)	0.67	0.12	0.43-0.80
		C>T	26% (7/27)	1.41	0.22	1.21-1.83

**BRCA2**	rs144848	C>A	65% (24/37)	1.60	0.55	1.24-3.62

**C1qA**	rs172378	A>G	69% (11/16)	1.86	0.77	1.26-3.37
		G>T	29% (5/17)	0.72	0.06	0.67-0.83

**CCND3**	rs1051130	T>G	24% (4/17)	1.36	0.11	1.25-1.51
		C>A	5% (2/37)	0.79	0.01	0.78-0.80

**EMSY**	rs228611	A>C	5% (2/37)	1.38	0.21	1.23-1.53

**GPX1**	rs1050450	C>T	79% (11/14)	1.32	0.09	1.23-1.50

**GPX4**	rs713041	C>T	100% (21/21)	6.46	2.97	2.5-11.31

**MLH3**	rs175080	NA	0% (0/35)	NA	NA	NA

**MTHFR**	rs1801133	T>C	82% (27/33)	0.65	0.06	0.54-0.77

**NBS1**	rs709816	C>T	14%(3/21)	0.82	0.01	0.81-0.83
		T>C	33% (7/21)	1.37	0.19	1.21-1.65

**TP53**	rs1042522	C>G	100% (16/16)	0.34	0.06	0.25-0.49
		T>C	17% (5/29)	0.75	0.06	0.66-0.82

**TRXR2**	rs1139793	C>T	31% (9/29)	1.38	0.13	1.21-1.61

DAE = differential allelic expression.

We found considerable variation in the magnitude of DAE across genes, with the largest seen in *GPX4 *(approximately six-fold difference between the levels of expression of the two alleles). For the genes which show DAE in at least one heterozygote, the proportion of heterozygotes with unequal expression ranged from 10% to 100% (Table 2). Genes with cis-acting elements in LD with the tSNP showed a direct correlation between mean allelic ratio and number of heterozygotes with variation (that is, the greater the mean allelic ratio, the higher the number of heterozygotes with DAE) [see Additional file 1]. Genes with cis- regulation not in LD with the tSNP had a distribution of ratios that was commonly centred on the 50:50 ratio (log_2 _= 0 in Figure 1a). This reflects the fact that a proportion of the heterozygotes for the tSNP will be homozygote for either of the regulatory polymorphic alleles, consequently generating an equimolar transcription level of the tSNP alleles.

Since peripheral blood is a heterogeneous tissue, composed of mononuclear cells (including B lymphocytes), polymorphonuclear cells and red blood cells, we compared the allelic expression ratios in cDNA extracted from total mononuclear cells from 59 healthy unrelated donors, with those obtained for sorted B cells. We found no significant differences in terms of pattern (cis- regulation in LD with tSNP or not) or mean ratio of DAE (data not shown).

Concerns have also been raised about the effect that transformation of lymphoblasts by Epstein-Barr Virus (EBV) may have on their expression profile [32-34]. As most previous studies of differential allelic expression have been performed on lymphoblastoid cell lines (LCL) and future case-control studies using DAE could be performed on this type of sample, we next compared transformed and non-transformed lymphoblasts from 19 unrelated CEPH (Centre D'Étude du Polymorphism Humaine) individuals, who were heterozygous for most of the genes included in this study (black data points in Figure 1a). Eight out of 12 genes showed DAE in both transformed and fresh lymphocytes (*BRCA2, CCND3 *and *GPX1 *did not show DAE in LCL samples, in contrast to that observed in untransformed blood, whilst *MLH3 *showed the opposite). Of the eight genes which showed DAE, five presented mean ratios and patterns of allelic preferential expression that were comparable between the two samples sets (Figure 1a and Tables 3 and 4). *BRCA1*, *EMSY *and *NBS1 *showed significantly different results from those obtained for fresh blood, in terms of the mean fold difference between alleles and/or patterns of DAE (cis- regulation in tight LD or not).

**Table 3:** Comparison of differential allelic expression between breast and blood (fresh and transformed)

Gene	SNP	Informative Heterozygotes			Wilcoxon rank sum test p- value		
			
		Blood	LCL	Breast	Blood vs LCL	Blood vs Breast	LCL vs Breast
**BRCA1**	rs799917	27	13	6	4.40E-06	0.011	0.016
**BRCA2**	rs144848	37	6	8	NA	0.744	NA
**C1qA**	rs172378	16	5	7	0.076	NA	NA
**CCND3**	rs1051130	17	12	7	0.207	0.266	0.090
**EMSY**	rs228611	37	7	13	2.26E-05	0.029	0.047
**GPX1**	rs1050450	14	4	12	NA	NA	NA
**GPX4**	rs713041	21	8	5	0.366	0.057	0.091
**MLH3**	rs175080	35	12	13	NA	NA	0.772
**MTHFR**	rs1801133	33	7	12	0.030	1.53E-04	0.703
**NBS1**	rs709816	21	9	13	0.003	0.001	0.947
**TP53**	rs1042522	16	8	16	0.192	1.34E-05	3.80E-04
**TRXR2**	rs1139793	29	9	14	0.069	0.826	0.007

Wilcox rank sum tests were performed to compare deviations of differential allelic expression variance (only genes showing DAE were introduced in the analysis). *Blood *corresponds to fresh B cell samples and *LCL *to EBV transformed lymphoblastoid cell lines. DAE = differential allelic expression; LCL, EBV transformed lymphoblastoid cell lines.

**Table 4:** Differential allelic expression concordance between breast and blood (fresh and transformed).

	Blood vs LCl	Blood vs Breast	Breast vs LCL
DAE present in both	8/12	9/11	10/11
Similar DAE distribution/pattern	5/8	8/9	9/10

Number of genes concordant in terms of presence and extent/pattern of preferential allelic expression. DAE = differential allelic expression; LCL, EBV transformed lymphoblastoid cell lines.

### Comparison of DAE between breast tissue and blood cells

Next, we analysed 40 normal breast tissue samples (green data points in Figure 1a). Like blood, breast is a complex organ, comprising breast epithelium, stroma, and adipocytes. The comparison between fresh blood and breast tissue showed that DAE distributions were similar for eight out of nine genes (89%) that showed DAE in both tissues. *BRCA1, BRCA2, CCND3, EMSY, GPX4*, and *TRXR2 *had similar mean ratios (based on Wilcox rank sum test) and/or patterns. In breast samples, the same alleles of *MTHFR *and *TP53 *were preferentially expressed as in the fresh blood samples, although with significantly different mean ratios (Tables 2 and 3). *GPX1 *showed no DAE in breast and *MLH3 *showed no DAE in blood, whilst *NBS1 *showed discordant patterns and mean allelic ratio.

Comparing the results obtained for transformed lymphoblasts with those obtained for breast we found that 10 out of 11 genes showed preferential allelic expression in both types of sample. Of these 10, five genes were comparable in terms of pattern and mean allelic ratio (*CCND3, GPX4*, *MLH3, MTHFR *and *NBS1*), and four were comparable only on pattern (*EMSY, TP53 *and *TRXR2*). Only *BRCA1 *was significantly different between the two sample sets for both mean allelic ratio and pattern of preferential expression.

Pairwise correlation analysis with the mean allelic ratios obtained for the genes that showed evidence of DAE in each two sample types showed high correlation across types of tissue (blood vs LCL R^2 ^= 0.88, blood vs breast R^2 ^= 0.80 and breast vs LCL R^2 ^= 0.87) (Figure 1b).

### Comparison between DAE analysis and linkage mapping of expression phenotypes

**Figure 2:**
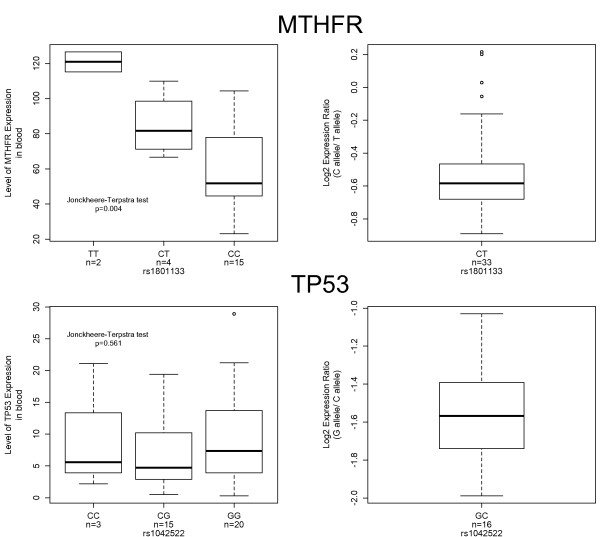
Comparison between identifying cis-regulatory elements by the total expression and the allelic expression ratio method in blood samples. For both genes, the graph on the left represents the correlation between the total level of expression and the genotype at the specified tSNP (*P*-values were calculated using the Jonckheere-Terpstra test). The graphs on the right-hand side represent the log_2 _ratios of allelic expression in heterozygotes only, for the corresponding tSNPs.

For the genes that in blood showed evidence for regulation from within the same linkage disequilibrium block (that is, genes for which all heterozygotes with imbalances showed preferential expression of the same allele), we determined total levels of expression using Taqman technology, for individuals of all genotypes. After, we performed a correlation analysis of the total level of expression vs genotype at the tSNP for these genes. We found that only *MTHFR *showed a significant correlation (*P *< 0.005) (Figure 2). For other genes, for example *TP53*, we found that total expression did not vary with genotype, even though we found evidence for differential allelic expression in our initial analysis [see Additional file 2].

## Discussion

Here we report an extensive analysis of differential allelic expression in breast and blood (fresh and EBV-transformed) in a set of candidate breast cancer genes using a large set of unrelated individuals of European origin. We demonstrate the feasibility of using DAE in fresh blood or transformed lymphoblasts as a quantitative trait in future association studies for susceptibility to breast cancer, as well as an approach to select genes/loci from the lists produced by the genome-wide association studies for further functional investigation and validation. We found that the magnitude (fold difference) or pattern (direction) of differential expression was concordant in eight out of nine genes which showed DAE in breast and fresh blood. The results were similar between in fresh and transformed lymphoblasts.

As reviewed by Williams *et al*. [35], the percentage of genes reported to be affected by genetic variation at cis-acting regulatory elements differs greatly between approaches. The most common approach to studying variation in gene expression has been linkage analysis of total gene expression [13,15,17], which in general reports 1 to 20% cis-linkages. When using imbalances of allelic expression in heterozygotes, previous reports point to a much greater proportion of genes (30 to 60%) with cis-acting regulation [14,16,18,36-39]. The discrepancy of proportion of genes showing cis-regulation reported by the different methods, that we also observe in our study (for *TP53 *for example), is in our view possibly the effect of a feedback control loop that maintains the total level of expression at a constant level inside the cell, irrespective of the genotype at the regulatory element. A major advantage of studying DAE is that as allelic transcript levels are compared within the same cellular and haplotype context, environmental factors, including the level and availability of transcription factors, and genetic biases are eliminated increasing the ability to detect the cis-effects (reviewed in [40,41]). However, the high percentage of DAE that we observe in out study is likely to be biased by our list of candidate genes, and will not necessarily correspond to the percentage of DAE genome-wide for any of the tissues we studied.

The previous studies that have looked at DAE in multiple tissues have reported significant differences for one gene examined in 12 individuals [25], and for 11% of 92 studied genes in six mice [26]. Our findings differ from these for two possible reasons: we have increased statistical power due to the larger number of samples analysed compared with the Wilkins et al study [25], but also because we analysed a smaller number of genes than Campbell et al [26].

We show that the difference between allelic expression levels can vary greatly (up to six-fold) across genes and based on previous reports [14,15] we assume that the distribution pattern of DAE can shed light on the nature of the regulatory cis-element causing DAE [7,16]. In addition, we note that the proportion of heterozygotes displaying DAE can differ greatly between genes (11% to 100% of heterozygotes). In only a small number of genes did all heterozygotes show preferential expression of one allele (two genes in all three sample sets, and two others in transformed lymphoblasts alone). In general, high mean allelic ratios correlated with a high proportion of samples with DAE. This suggests that regulatory variants have in fact non-binary, stochastic effects on the binding of transcription factors. If in some cases the effect is a very strong one, consequently more heterozygotes will present it. For example, in the case of a polymorphism that alters the affinity of binding of a transcription factor [6,20], the extent of the effect we detect is probably a reflection of where on the binding site sequence the nucleotide change occurs, and how specific the binding of the transcription factor is to a certain sequence. All of these considerations become important when carrying out haplotype analysis to map which regulatory variants are mechanistically responsible for DAE.

Overall, our results suggest that although the total level of expression of a gene is under tissue-specific regulation (mainly due to the availability of the necessary transcription factors), DAE is mostly tissue-independent -exerting a similar effect in most tissues where the gene is expressed - and individual specific - regulated by the genetic variation make-up of each individual (even in the same cellular/tissue context). However, it is likely that tissue-specific levels of transcription factors might also influence the magnitude of DAE, as we noted in genes that show evidence of being regulated differently in the studied tissues (*BRCA1 *in B cells and breast, for example). Ideally, for validation, this study should be followed-up with another on matched blood and breast samples.

## Conclusions

In conclusion, we show that differential allelic expression is common in candidate breast cancer genes and is comparable between tissues to some extent. Our findings support the further exploration of DAE in blood and breast as a quantitative phenotype to reveal regulatory genetic variation that predisposes to breast cancer (as in recent reports for breast and colorectal cancers [9,42]), as well as a mean to prioritise the candidate susceptibility hits from the GWAS for follow-up functional studies and confirmation.

## Abbreviations

CEPH: Centre D'Étude du Polymorphism Humaine; DAE: differential allelic expression; EBV: Epstein-Barr virus; GWAS: genome-wide association studies; LCL: lymphoblastoid cell line; LD: linkage disequilibrium; SNP: single nucleotide polymorphism; tSNP: transcribed/transcript single nucleotide polymorphism.

## Competing interests

The authors declare that they have no competing interests.

## Authors' contributions

ATM conceived and designed the study, prepared samples, carried out experiments, prepared and edited the manuscript. IS carried out sample preparation and performed experiments. AJXL performed experiments. LJ carried out sample collection and elaborated ethics applications. MOR contributed to sample preparation. CC and BAJP helped conceiving the study and editing the manuscript. All authors have read and approved the final manuscript.

## Supplementary Material

Additional file 1 Adobe Acrobat document containing a graph of the correlation between mean ratio of DAE and percentage of heterozygotes with DAE in B cells. Click here for file

Additional file 2 Adobe Acrobat document containing the graphs for all extra genes in the total level of expression vs genotype correlation analysis. *P *values correspond to the Jonckheere-Terpstra test, like for Figure 2. Click here for file
