# Supplementary figures and images for: Extent of differential allelic expression of candidate breast cancer genes is similar in blood and breast
Source: Breast Cancer Res. 2009 Dec 10;11(6):R88. doi: 10.1186/bcr2458 (PMC2815552; doi:10.1186/bcr2458)

Percentage of Heterozygotes  
showing DAE

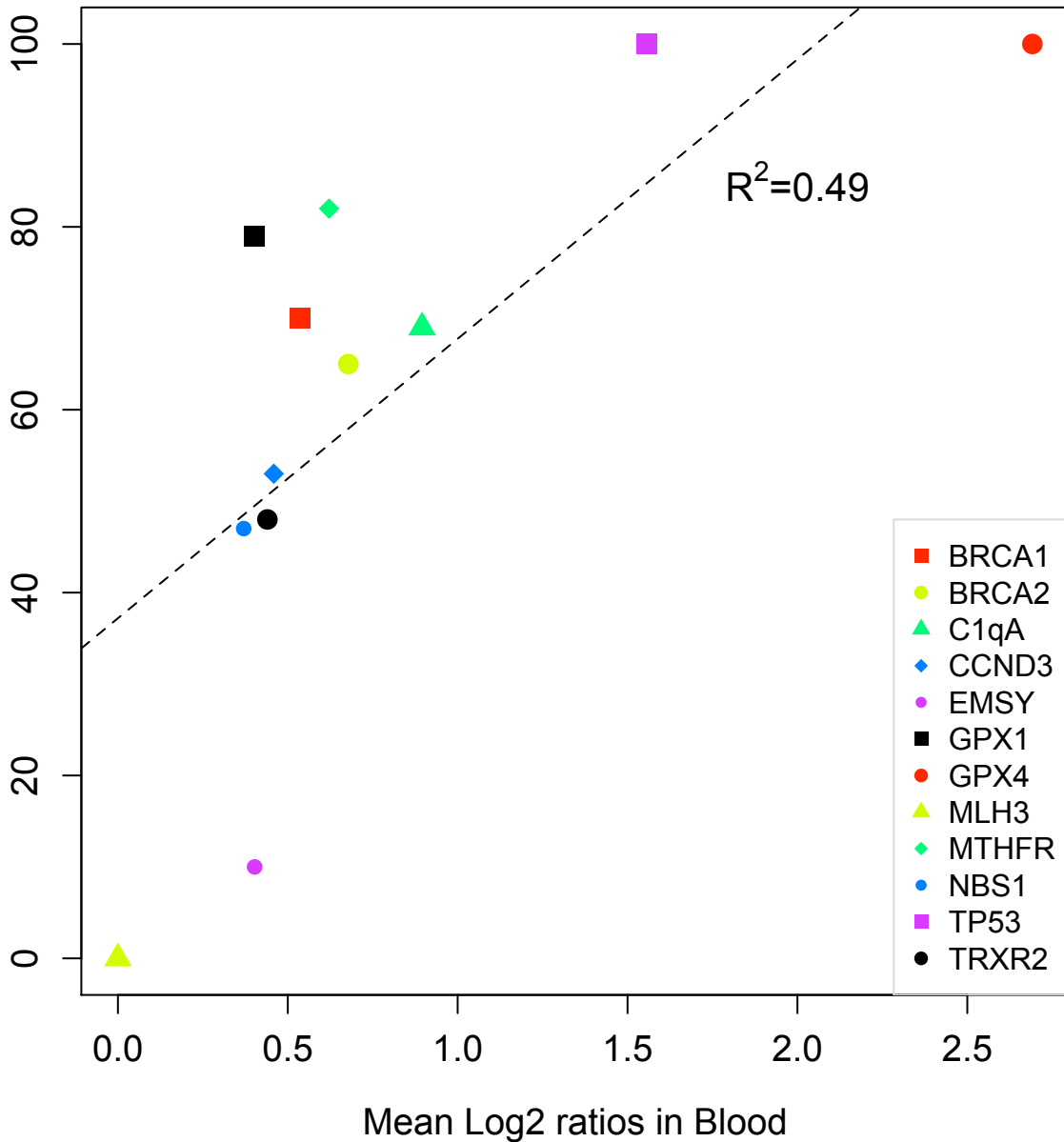

Supplement: Additional file 1 — Adobe Acrobat document containing a graph of the correlation between mean ratio of DAE and percentage of heterozygotes with DAE in B cells. [file bcr2458-S1.pdf]

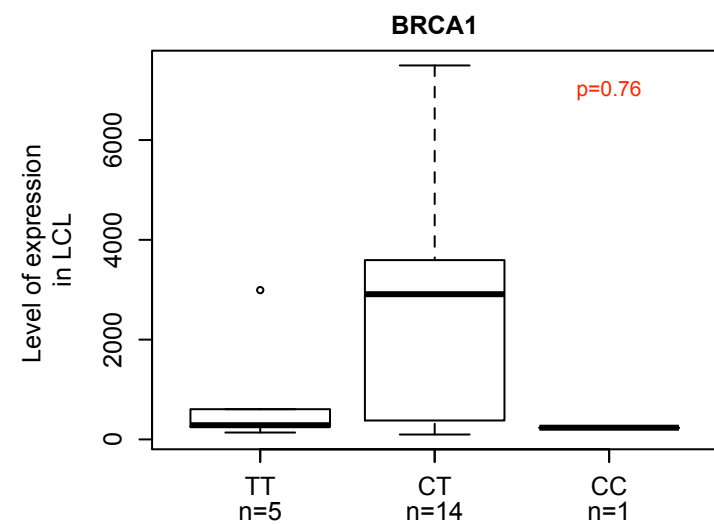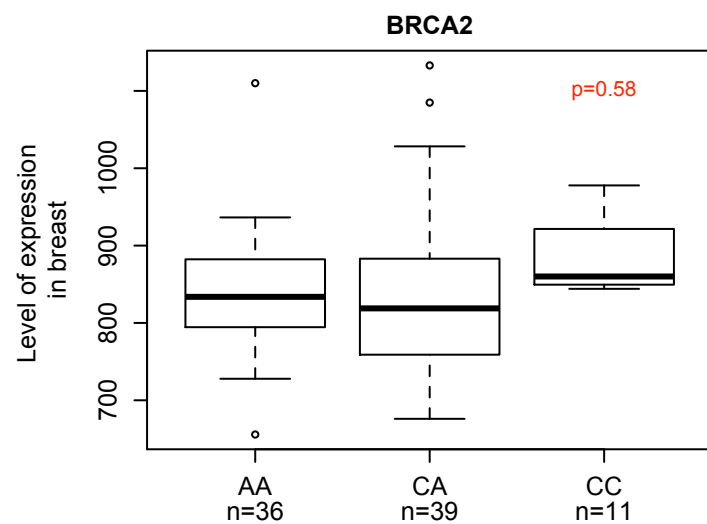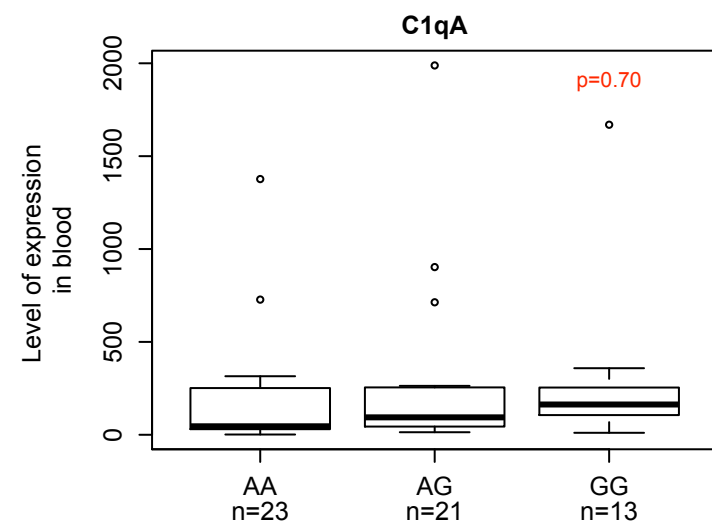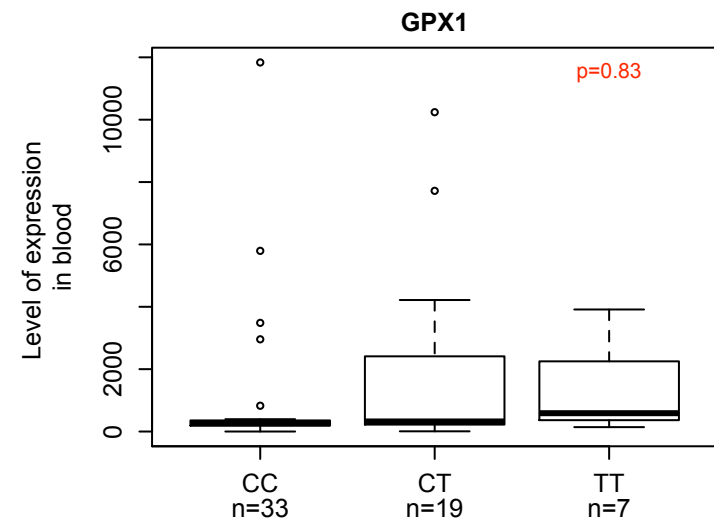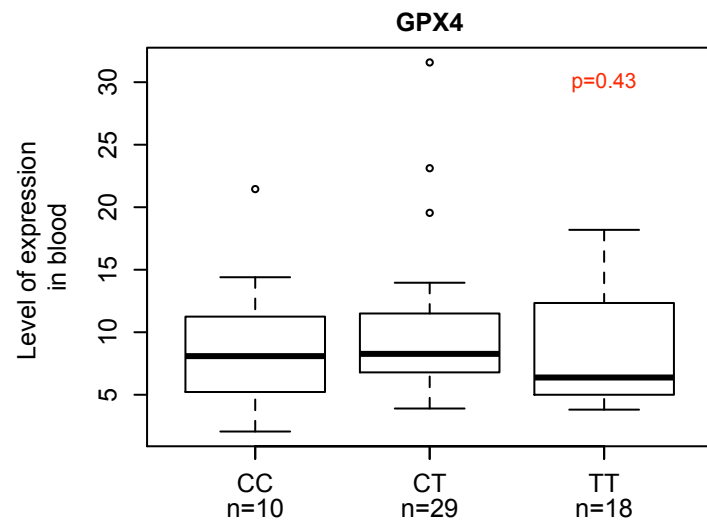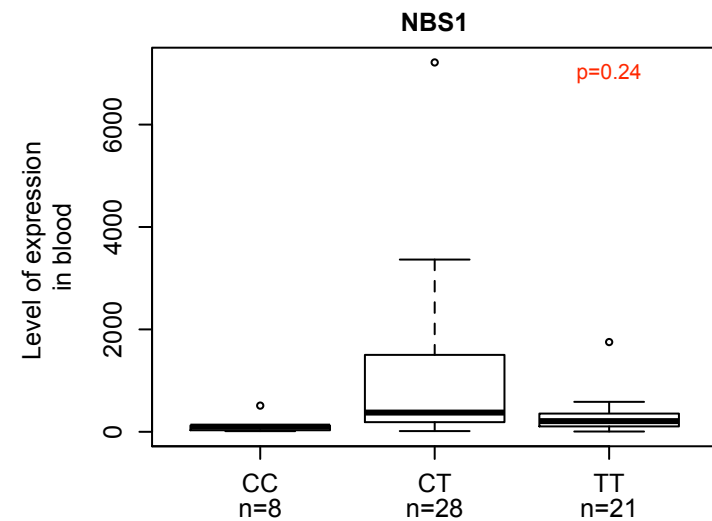

Supplement: Additional file 2 — Adobe Acrobat document containing the graphs for all extra genes in the total level of expression vs genotype correlation analysis. P values correspond to the Jonckheere-Terpstra test, like for Figure 2. [file bcr2458-S2.pdf]
